# Impact of severe polyhandicap on parents’ quality of life: A large French cross-sectional study

**DOI:** 10.1371/journal.pone.0211640

**Published:** 2019-02-04

**Authors:** Marie-Christine Rousseau, Karine Baumstarck, Sherezad Khaldi-Cherif, Catherine Brisse, Agnès Felce, Benjamin Moheng, Anderson Loundou, Thierry Billette de Villemeur, Pascal Auquier, the French Polyhandicap Group

**Affiliations:** 1 Fédération des Hôpitaux de Polyhandicap et Multihandicap, Hôpital San Salvadour, Assistance Publique Hôpitaux de Paris, Hyères, France; 2 EA 3279, Self-perceived Health Assessment Research Unit, School of Medicine, Aix Marseille Université, Marseille, France; 3 Union Générale Caisse Assurance Maladie (UGECAM), Paris, France; 4 Comité d'Études, d'Éducation et de Soins Auprès des Personnes Polyhandicapées, Paris, France; 5 Hôpital d’Hendaye, Assistance Publique Hôpitaux de Paris, Hendaye, France; 6 Sorbonne Université, UPMC, GRC ConCer-LD, Paris, France; 7 Service de Neuropédiatrie—Pathologie du développement, Centre de référence des déficits intellectuels de causes rares, Hôpital Trousseau, Assistance Publique Hôpitaux de Paris, Paris, France; 8 Service de Polyhandicap Pédiatrique, Assistance Publique Hôpitaux de Paris, Hôpital de La Roche Guyon, La Roche Guyon, France; Iranian Institute for Health Sciences Research, ISLAMIC REPUBLIC OF IRAN

## Abstract

**Background:**

Polyhandicap (PLH) is a condition of severe and complex disabilities and is defined by a combination of profound intellectual impairment and serious motor deficits. Parents of PLH individuals are chronically confronted with stressful situations. The aims of this study are i) to assess and compare the quality of life (QoL) of a large panel of parents of PLH individuals with age- and gender-matched controls and ii) to identify potential determinants of parents’ QoL.

**Method:**

We conducted a cross-sectional study. Parents were recruited from 4 specialized rehabilitation centres, 9 residential facilities, and a specialized paediatric/neurological department. The selection criteria were age above 18 years and being the mother/father of a PLH individual. The data collected from the parents included sociodemographic, health status, and psycho-behavioural data (including QoL); additionally, the health status of the PLH individuals was collected.

**Results:**

The QoL scores of all dimensions were significantly lower for parents than for controls. The main factors modulating parents’ QoL were financial issues, health status, and coping strategies. The PLH individuals’ health status was not associated with parents’ QoL.

**Conclusions:**

Some QoL determinants might be amenable. These findings should help health care workers and health decision makers to implement specific and appropriate interventions.

## Introduction

Polyhandicap (PLH), as a recently defined concept [1–3], it is a dramatic health condition comprising severe and complex disabilities corresponding to a chronic disorder occurring in an immature brain, leading to a combination of a profound intellectual impairment and a serious motor deficit and resulting in an extreme restriction of autonomy and communication. PLH is close to profound intellectual and multiple disabilities (PIMD), but PLH does not systematically refer to a disorder affecting an immature brain [4]. Due to the entanglement of various heavy handicaps and multiple comorbidities [5], the patients need permanent human and technical assistance throughout their life.

In this context, the families of the PLH individual, from birth or the first years of life of the child, are repeatedly and chronically confronted with stressful situations: the announcement of such a diagnosis and poor prognosis, the understanding of the neurodevelopment status, the complexity of future projection, and the potential occurrence of sudden health deterioration, etc. It is riskless to hypothesize that the parents, due to the singular link they have with the PLH individual, are the most affected. This particular life event, which leads to a major lifestyle disruption for both mothers and fathers [6], requires parents of individuals to permanently develop and mobilize significant internal and external resources [7]. Due to the improvement of life expectancy, the progressive move from traditional institutional care to deinstitutionalization care, and a (slow) reduction of stigmatization, the burden of care is gradually transferred to family caregivers of individuals with disabilities or loss of autonomy, who now partially assume new functions. Despite being surrounded by logical and human assistance, the impact of PLH on the social, psychological, and physical conditions, and consequently on the quality of life (QoL) of parents is considerable. While some studies have explored the QoL of parents who have children with cerebral palsy [8], few data are available for parents of individuals with PIMD [9,10]. Despite the acknowledged need to consider caregiver experience issues, there are no data about caregivers of PLH individuals. However, assessment of parents’ QoL and knowledge of which factors are determinants of this QoL would strongly assist clinicians and health decision making authorities in offering appropriate interventions: human aid, technical aid, respite care, and psychological support.

The aims of this study were as follows: i) to assess the QoL of a large French panel of parents of PLH individuals and to compare their QoL with French age- and gender-matched controls; ii) to identify potential determinants of parents’ QoL and iii) to identify the association between parents’ QoL and social environment and satisfaction with the healthcare system.

## Materials and methods

### ■ Design and settings

This study incorporated a cross-sectional design. The recruitment of parents was made from 4 specialized rehabilitation centres, from 9 residential facilities of the French Comité d'Études, d'Education et de Soins Auprès des Personnes Polyhandicapées Association (CESAP), and from a specialized paediatric/neurological department of a university hospital (Service de Neuropédiatrie, UPMC, Hôpital Trousseau, Assistance Publique Hôpitaux de Paris, France). This study was included in the French national PoLyHandicap (PLH) cohort (see above).

### ■ General organization of the PoLyHandicap cohort

The general aim of the French PLH cohort was to identify the impact of potential (socioeconomic, environmental, epidemiologic) determinants on the health status of the individuals and the daily life of their (natural and institutional) caregivers (NCT02400528). Three different populations were eligible: i. individuals with severe PLH defined by the combination of motor deficiency (tetraparesis, hemiparesis, paraparesis, extra pyramidal syndrome, cerebellar syndrome, neuromuscular problems) and intellectual impairment (Intelligence quotient IQ <40), associated with everyday life dependence (functional independency measure FIM <55), restricted mobility (Gross Motor Function Classification System levels GMFCS III, IV and V), and age at onset of cerebral lesion below 3 years old; ii. institutional health care workers of the included individuals; iii. familial referents of the included individuals (French legal mention for this kind of individuals, represented by parents in most cases). The present study focused on parents as familial referents.

### ■ Selection criteria

The selection criteria were as follows: aged above 18 years; being an official familial referent (mother and/or father) of a individual included in the PLH cohort; and agreeing to participate. The exclusion criteria were: being another familial caregiver than mother/father of the PLH person, refusal to participate.

### ■ Data collection

Two sources of data were used: the medical records and the parents themselves.

- From the medical records, characteristics of the individual were collected: age, gender, place in the sibling (elder or not), global health severity (severe for individuals who meet all the following criteria: motor handicap (paraparesia or tetraparesia and/or extrapyramidal syndrome and/or severe general hypotonia), IQ <25, FIM ≤20, and GMFCS IV and V; less severe for individuals who do not meet these criteria); global health stability (unstable for individuals who meet at least one of the following criteria: recurrent pulmonary infections (≥5/yrs), drug-resistant epilepsia (≥4 seizures/month); stable for individuals who do not meet any of these criteria), medical devices (at least one of the following list: invasive mechanical ventilation, non-invasive mechanical ventilation, tracheotomia, nasogastric tube, gastrostomy, permanent urinary probe, cerebrospinal fluid derivation, and central venous catheter).
- The data collected from the parents themselves were gathered into a booklet. The booklet included the following data:
  1. Sociodemographics: nature of the relationship with the PLH individual (mother/father), age, marital status (not single/single), in couple with the other parent of the PLH individual, number of children living at home and notion of another handicapped person living at home, educational level, occupational status (worker/not worker), self-perceived financial status, importance of the presence of the PLH individual at home (<7/> = 7 nights/month),
  2. Health and presence and nature of chronic diseases; hospitalization episode during the last 2 years; other health resources use during the past 3 months (anxiety and/or stress medications, psychological support, alternative medicines).
  3. Psycho-behavorial data: Anxiety-mood disorders assessed using a score ranged from 1 (absence) to 10 (very important). The coping was assessed using the Brief Coping Orientation to Problems Experienced Scale (Brief-COPE), exploring 4 dimensions that include social support, problem solving, avoidance, and positive thinking [11]. Scores ranged from 0 to 100. High scores reflect a high tendency to implement the corresponding coping strategies.
  4. Specific questions were proposed regarding: i) the social environment: family relationship preservation, presence of the PLH individual during family celebrations, existence of a social network related to the PLH/not related to the PLH, PLH associative community implication. ii) the parents’ healthcare satisfaction: medical information related to the PLH individual, global management information quality, quality of care provided to the PLH individual, family caregivers services provided.
  5. Quality of life was assessed using the World Health Organization Quality of Life (WHOQOL-BREF) questionnaire which is a generic questionnaire used worldwide [12]. It describes four domains: physical health, psychological health, social relationships, and environment. All scores range between 0 and 100, with higher scores indicating a better QoL. French norms are available for three domains [12].

### ■ General procedure

For each included PLH individual, data were collected from medical records obtained by a dedicated clinical research assistant and was supported by the referent physician of the PLH individual (a referent physician is designated for each individual). A maximum of 4 official familial referents were systematically identified in the medical record. The familial referents are the resource persons who should be contacted for any medical, administrative, and social issues. From March 2015 to December 2016, the booklet was sent by mail to each mother and each father identified as a referent person. To optimize the participation, a prepaid return envelope addressed to the coordination team was added to the mail to optimize the participation.

### ■ Ethics approval and consent to participate

Regulatory monitoring was performed according to the French law that requires the approval of the French ethics committee (Comité de Protection des Personnes Sud Méditerranée V, approval: 20/10/2014, reference number 2014-A00953-44). A written consent form was obtained for each participant.

Trial registration: **NCT02400528**. Registered 3 February 2015.

### ■ Statistics

The WhoQoL scores of familial caregivers were compared to those obtained from French age- and gender-matched controls from a healthy sample of 16 392 subjects [12]. Comparisons of QoL scores between different subgroups (parents’ variables: nature of the relationship with the PLH individual, marital status, initial couple of parents, other handicapped person at home, educational level, occupational status, financial status, monthly presence of the PLH individual at home, chronic disease(s), hospitalization; PLH individuals’ variables: gender, rank in the sibling, severity, stability, medical devices) were performed using Student’s t tests. Associations between QoL scores and continuous variables (age of parents, anxiety-mood score, coping scores, age of children) were analysed using Pearson’s correlations. Multivariate analyses using Generalized Estimating Equations models were performed to identify variables linked to QoL scores. In the models, each QoL dimension score was considered to be a separate dependent variable. The independent variables relevant to the models were selected from the univariate analysis, based on a threshold *P*-value of ≤0.20. The final models produced standardized beta coefficients, which represent a change in the SD of the dependent variable (QoL score) resulting from a change of one SD in the various independent variables. Independent variables with higher standardized beta coefficients are those with a greater relative effect on QoL. To consider the nature of the relation of participant with the PLH individual, the same procedure was independently performed for mothers and fathers. Statistical analyses were performed using SPSS software (IBM SPSS PASW Statistics Inc., Chicago, Ill USA). All tests were two-sided. The threshold for statistical significance was set at *P* <0.05.

## Results

A total of 1273 questionnaires were proposed to referent parents of PLH individuals included in the cohort. During this period, 394 parents (response rate: 31%), corresponding to 295 PLH individuals, returned the questionnaires. For 69 PLH individuals, both parents (138) participated. Among the 879 non-responders, a 10% random sample (n = 87) was called by phone in order to collect a reason of non-participation: difficulty understanding French language (n = 29), being unable to talk about this experience (n = 23), refusal to give any reason (n = 17), personal health difficulties (n = 3), and 15 parents were not reachable. The PLH individuals’ characteristics of the 879 non-participants did not differ from the PLH individuals’ characteristics of the 394 participants in terms of gender, severity, stability, and presence of devices; the participants had younger children in comparison with the non-participants (19.7+/-14.6 vs. 27.0+/-17.4, p<0,05).

### ■ Characteristics of the sample

All the characteristics of the parents are provided in Table 1. Participants were aged from 19 to 85, and 64% of them were mothers. At inclusion, two-thirds of the participants were still in a relationship with the second parent of the PLH individual. Thirteen percent of participants reported living with a second handicapped person. One-third of the respondents reported at least one chronic disease: 43 (11%) musculoskeletal trouble, 31 (8%) cardiovascular disease, 21 (5%) diabetes, 16 (4%) asthma, 5 thyroid problems, 5 cancer, and 26 other diseases. Twenty percent of parents reported a hospitalization episode during the last 2 years. The coping strategies that were based on social support and avoidance were less common than strategies based on problem solving and positive thinking. The characteristics of the corresponding PLH individuals are detailed in Table 1.

**Table 1:** Characteristics of the sample.

Parents		N = 394
**1. Sociodemographics**		**N (%)**
Age (years)	Mean±SD	49.1±12.7
Nature of relationship	Mother	250 (63.7)
	Father	142 (36.3)
Marital status	Not single	288 (73.5)
	Single	104 (26.5)
Couple with the other parent of the PLH individual	Yes	242 (62.9)
	No	143 (37.1)
Number of children living at home	Mean±SD	2.2±1.1
Other handicapped person living home	Yes	51 (13)
	No	338 (87)
Educational level	<12 years	187 (48.3)
	≥12 years	200 (51.7)
Occupational status	Not worker	181 (46.6)
	Worker	208 (53.4)
Self-perceived financial status	Not difficult	286 (75)
	Difficult	100 (26)
Presence of the PLH individual at home	<7 nights/month	174 (44.3)
	≥7 nights/month	219 (55.7)
**2. Health**		N (%)
Personal chronic disease(s)	Yes	122 (31.4)
	No	266 (68.6)
Hospitalization episode during the last 2 years	Yes	77 (19.9)
	No	310 (80.1)
**3. Psycho-behavorial characteristics**		N (%)
Anxiety-mood score (1–10)*	Mean±SD	5.7±2.7
Coping strategies (BriefCope scores)**	Social support	39.3±21.7
	Problem solvings	58±23.5
	Avoidance	23±12.6
	Positive thinking	53.1±19.7
**PLH individuals**		**N = 295**
Age (years)	Mean+/-SD Med [IQR]	19.7+/-14.5 16 [9–26]
Gender	Girl/Woman	132 (44.7)
	Boy/Man	163 (55.3)
Severity***	Less severe	133 (45.5)
	Severe	159 (54.5)
Stability****	Stable	210 (71.9)
	Unstable	82 (28.1)
Medical devices*****	Yes	111 (37.8)
	No	183 (62.2)

* 1 absence to 10 very important disorder

** high score, high tendency to implement the strategies

*** Severe case: association of motor handicap, IQ <25, FIM< = 20, and GMFCS IV/V

**** Unstable case: recurrent pulmonary infections and/or drug resistant epilepsy

***** at least one of the following list: invasive mechanical ventilation, non-invasive mechanical ventilation, tracheotomia, nasogastric tube, gastrostomy, permanent urinary probe, cerebrospinal fluid derivation, and central venous catheter

### ■ Quality of life

**Fig 1:**
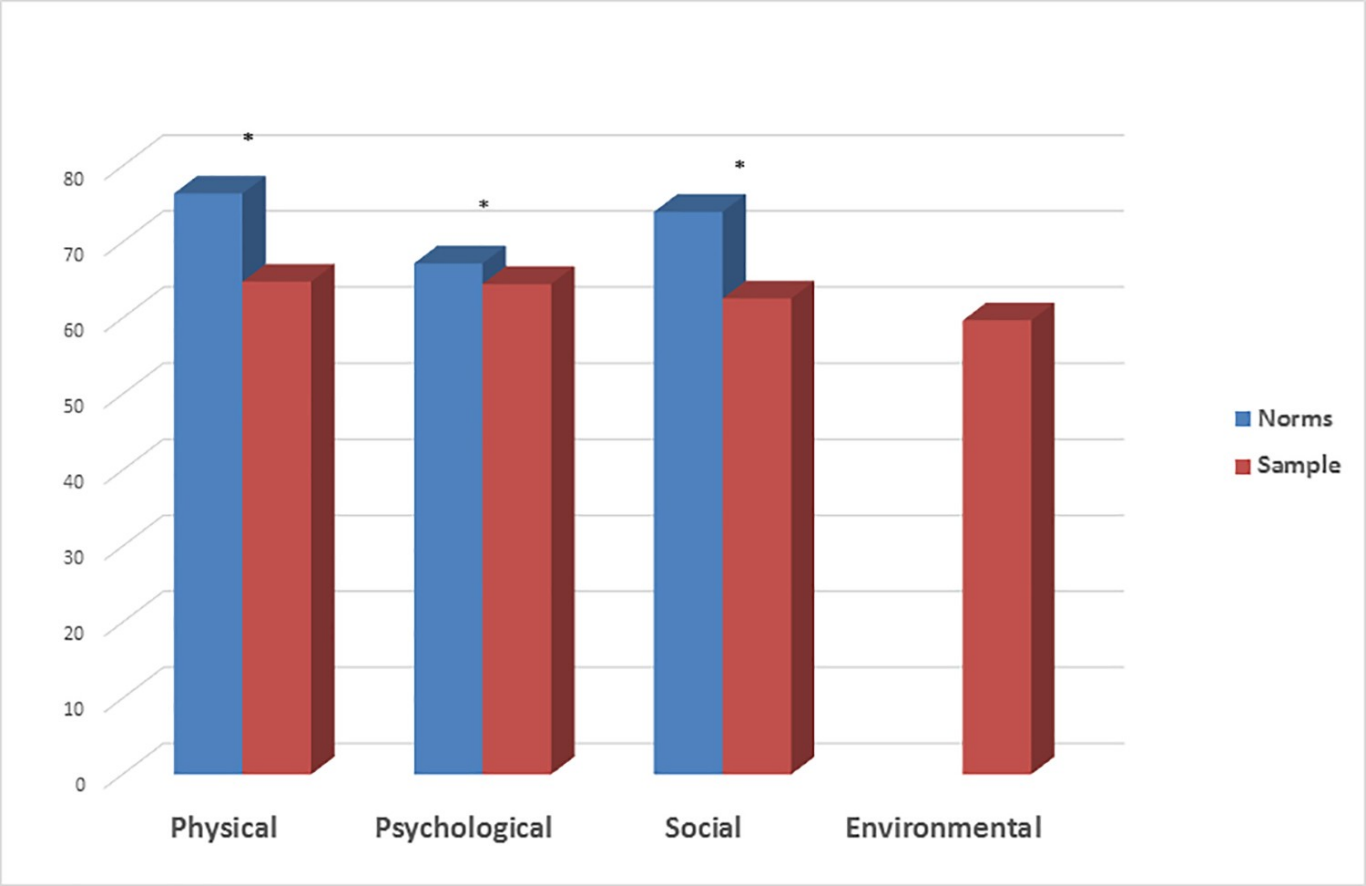
Comparisons of WhoQoL scores between the familial caregivers and French age sex matched norms. Higher the scores.higher the QoL level° Baumann C, Erpelding ML, Regat S, Collin JF, Briancon S: The WHOQOL-BREF questionnaire: French adult population norms for the physical health, psychological health and social relationship dimensions. RESP 2010, 58(1):33–39. * p<0.05.

The QoL scores of the parents are provided in Fig 1. Of the 3 dimensions of the WhoQoL for which French norms are available (physical, psychological, and social dimensions), the participants reported significantly lower scores compared with age- and sex-matched controls.

### ■ Factors modulating the quality of life of parents

The results for the factors modulating the QoL of the parents from the univariate analysis are provided in additional material (Table A and Table B in S1 File). After adjustment, the factors modulating the QoL were as follows:

1. Alteration of physical QoL dimension: reporting financial difficulties, being not worker, having a chronic disease or reporting a recent hospitalization episode, anxiety-mood disorders, and insufficient use of problem solving as coping strategy. 2. Alteration of psychological QoL dimension: being the mother (in comparison with the father), reporting financial difficulties, anxiety-mood disorders, use of avoidance and non-use of the 3 other coping strategies (social support, problem solving, and positive thinking); 3. Alteration of the social QoL dimension: having the PLH individual more than 7 nights per month at home, having a chronic disease, anxiety-mood disorders, non-use of social support and problem solving as coping strategy, and having a male PLH individual in comparison with having a female PLH individual; 4. Alteration of the environmental dimension: having a lower educational level, reporting financial difficulties, having the PLH individual more than 7 nights per month at home, having a chronic disease, anxiety-mood disorders, and non-use of problem solving as coping strategy. Some factors were not linked to the parents’ QoL: age of the parents, their marital status, the relationship status with the second parent of the PLH individual, having children, and all the characteristics of the PLH individuals such as age of the PLH individual and place in the sibling, and indicators of health status such as severity, stability, and presence of devices. All the details are provided in Table 2.

**Table 2:** Factors modulating parents’ quality of life (using Generalized Estimating Equations models).

		Physical		Psychological		Social		Environ.	
		β	p-value	β	p-value	β	p-value	β	p-value
**1. Parents’ variables**									
Relation with the PLH individual (0 mother, 1 father)		1,486	0,365	-2,861	**0,036**				
Age of the parent								0,026	0,828
Marital status (0 single, 1 couple)								-2,822	0,126
Couple initial of parents (0 yes, 1 no)				0,154	0,92	2,724	0,182		
Educational level (0 low, 1 high)		2,171	0,187					3,769	**0,022**
Financial status (0 not difficult, 1 difficult)		-5,957	**0,013**	-6,955	**<10**^**−3**^	-2,282	0,339	-9,97	**<10**^**−3**^
Occupational status (0 worker, 1 not worker)		-6,853	**<10**^**−3**^	-1,434	0,343	-1,481	0,438	-2,034	0,23
Other handicapped person at home (0 no, 1 yes)		-3,728	0,129	-2,836	0,180			-4,249	**0,041**
Presence of the PLH individual at home (0 > = 7; < 7 nights)						9,91	**<10**^**−3**^	4,964	**0,03**
Chronic disease (0 no, 1 yes)		-11,216	**<10**^**−3**^	-0,695	0,642	-8,776	**<10**^**−3**^	-4,237	**0,009**
Hospitalization episode (0 no, 1 yes)		-6,928	**0,001**	-2,28	0,195	-2,642	0,244	-2,858	0,122
Anxiety-mood score		-2,245	**<10**^**−3**^	-2,036	**<10**^**−3**^	-2,229	**<10**^**−3**^	-1,026	**0,003**
Coping	Social support			0,096	**0,01**	0,214	**<10**^**−3**^		
	Problem solvings	0,148	**0,002**	0,137	**0,001**	0,101	**0,024**	0,188	**<10**^**−3**^
	Avoidance	-0,045	0,512	-0,254	**<10**^**−3**^				
	Positive thinking	0,019	0,676	0,143	**0,001**	0,092	0,08	-0,033	0,489
**2. PLH individuals’ variables**									
Gender of the PLH individual (0 boy/man, 1 girl/woman)				1,705	0,286	4,699	**0,016**	1,785	0,312
Age of the PLH individual						-0,033	0,711	0,098	0,457

β beta standardized coefficient; Bold values: p-value <0.05

Results were separately provided for mothers and fathers in the S1 Table. Two thirds of the tested links were concordant (significant or non-significant in the two sub-samples). Some links were found in the mothers’ sub-group but not for the fathers, partially due to a lack of power. No discordant results were found (significant in the two sub-samples with an opposite link).

### ■ Associations between parents’ quality of life and: social environment and satisfaction about the health care system

#### a) Social environment

A family relationship preservation (92%) and the presence of the PLH individual during family preservation (73%) improved parents’ QoL scores. Parents who reported having a social network not related to the polyhandicap field (74%) reported better QoL scores in all domains, whereas parents who reported having no social network that was not related to a social network related to polyhandicap (36%) reported lower physical scores. Implication in a PLH associative community (20%) did not impact QoL scores. All the details are provided in Table C in S1 File.

#### b) Satisfaction about the health care system

Parents reporting to be rather satisfied with medical information (56%), global management (64%), quality of care (68%), and family caregivers’ services (55%) globally scored better QoL. All the details are provided in additional material (Table C in S1 File).

## Discussion

It has been recognized that caregiving adversely affects the caregiver in terms of their health, emotional status, and quality of life [13–15]. While there is a vast literature assessing caregiving in various chronic/severe diseases (such as cancer [13,14], mental health diseases [15], various neurologic diseases [16,17], and in people with cerebral palsy [18–20], very few studies have assessed the impact of polyhandicap, which is a dramatic health condition leading to extreme physical and psychological dependency. This study explores, for the first time, the quality of life and factors modulating this QoL from a large sample of parents of PLH individuals.

The first interesting finding of our study is that the parents of PLH individuals reported a lower QoL for all the dimensions compared with the QoL of French age-gender matched controls. Some explanations should be given. For the physical impact, the complete dependence of the PLH individuals could lead to heavy and time-consuming care [6], and the institutionalization of the PLH individuals in specific care structures that may be far from home can require numerous tiresome trips [21]. The vast majority of our sample reported worrisome health indicators, such as a recent hospitalization episode (1/5) or chronic disease (1/3), including a large proportion of parents reporting musculoskeletal troubles, in line with similar studies [19]. These findings emphasize the need for technical/logistical/human aids to help familial caregivers to care for these very dependent PLH individuals. The social impact, which is also significant, should partially be explained by a persistent negative perception of polyhandicap in our societies. Even if progress may be seen, the PLH remains stigmatized due to various causes: the incurability of PLH, the dimorphic appearance of the PLH individuals (joint deformities, major scoliosis, and strange face), or the behavioural disorders that the PLH individual may develop. This stigmatization probably weighs on the families, leading to a self-restriction for roles and social activities. The psychological dimension of QoL has been examined by various studies demonstrating that caregiving increases psychosomatic, anxious, or depressive symptoms [19,22,23]. In line with these findings, substantial proportions of parents in our sample reported the regular use of anxiolytics or antidepressants and the need of psychological support. However, some issues should be discussed. First, the sample included parents of the most severe cases of PLH (due to the restrictive selection criteria), that may question about the representativeness of our results. However, our study provides findings from a homogeneous sample including severe individuals in comparison with similar studies which often assessed inhomogeneous sample including severe and less severe cases. Second, only QoL scores’ norms according to sex and gender are available. Scores according to health or socioeconomic status could reduce bias of the results. One of the difficulties encountered when interpreting a quality of life score for clinicians is the lack of norms values [24,25].

The second part of our findings refers to the QoL determinants. Identifying QoL determinants may help identify unmet needs, prioritize service improvements, and support funding decisions. The analyses that we performed showed that the main sociodemographic and socioeconomic parameters (such as age, gender, marital status, educational level, and occupational status) cannot be identified as significant QoL determinants as traditionally found in the literature. We only found ectopic (and expected) associations. The psychological domain was significantly lower for the women (i.e., the mothers of the PLH individuals) in comparison with the men (i.e., the fathers of the PLH individuals) [26,27]. Our separate analysis did not find important discordance between mothers and fathers. But it was often expected that mothers could be more affected by care giving of a child while fathers maintain some outside involvement. Future studies could specifically explore this assertion.

A higher educational level or having a job improved some aspects of QoL. While it is well known that working provides a social role and a salutary respite [23,26], only half of the parents reported having a job, which is much less than the general population (80%) [28] and less than parents of children with cerebral palsy (66%) [29].

More interestingly, the self-perceived financial situation was a strong determinant of QoL. The financial difficulties (reported by about one-quarter of the participants) may be the consequence of the combination of absence or unstable job (partially due to the time-consuming caregiving duties) and the necessary expenses for outdoor/indoor facilities or any specialized equipment (e.g., wheelchairs). These financial difficulties deserve attention from health authorities. Indeed, while various French financial aids are proposed to families, they are often confronted with administrative barriers and long lead-times. These administrative procedures should be simplified to meet the needs of the families. A more important presence of the PLH individual at home alters social and environmental domains, but paradoxically, this has no influence on physical and psychological aspects, probably due to respite care possibilities offered by many institutions. This result would suggest that a reinforcement of human aid for homecare may increase caregivers’ free time and improve some aspects of QoL.

The health status (chronic disease, hospitalization stay, anxiety, or mood disorders) of the parent was directly related to his/her self-reported QoL. Interestingly, we observed that the types coping strategies that individuals use may have a direct impact on their self-reported QoL. We found that individuals who used problem-solving, positive-thinking, or social support strategies reported higher QoL scores, while those who used avoidance strategies reported lower QoL scores. This finding encourages a more systematic assessment of coping styles to identify individuals who do not use healthy coping strategies and to offer targeted psychological interventions [30]. In other contexts, combined cognitive-rehabilitation and problem-solving therapy interventions have reported positive findings, and psychoeducation and cognitive behavioural therapy helped caregivers to maintain a stable QoL [17]. Therefore, taking care of the caregiver is a noteworthy issue that is proven to improve the caregiver’s role [31]. Future health-promotion strategies should be devised in order to prevent, diagnose, and treat any health-related problems of caregivers, including mental problems.

Surprisingly, we did not find an association between objective health indicators of the PLH individual (represented by severity or stability) and the parents’ QoL. This finding could be partially explained by the presence of a well-known phenomenon: ‘response shift’ or ‘adaptation to illness’ [32]. Similarly, we did not find an association between the age of the PLH individuals and the QoL of the parents. Here, again, we expected that parents would adapt to their child’s illness over time and thus report corresponding QoL changes. The onset of polyhandicap is a dramatic experience occurring early in the child’s life, and it leads the parents to mourn the loss of their child’s normalcy (their child who will never walk, speak, go to school and build a family, etc.). This difficult experience may overshadow all other health events (deterioration, instability, need of medical device, etc.), thus explaining the absence of an impact on the parents’ QoL compared with the QoL of parents of children with profound intellectual and multiple disabilities, which is a condition that carries more possibilities of development [6]. Longitudinal studies will help to better understand the functioning of the families and parents in particular.

More unexpectedly, the (social) QoL was lower for parents of a male individual than parents of female individual, and this link was independent of the gender (mother/father) of the parent. This finding refers to the social representation that people may develop. There is a long-standing and well-documented observation of male gender “preference” in developing countries [33]. Moreover, research from developed countries has found that having a boy may reinforce the marital stability or lead fathers to be heavily involved with caretaking[34]. Future studies should further explore these aspects using mixed approaches (quali-quantitative studies).

Other traditional socioeconomic indicators (such as health coverage, medical aid, or living area), were not assessed and should be taken into account in future studies. It has been shown that these variables may modulate self-reported QoL. This finding should be considered in the context of the national health care system. The French health care system tries to guaranty free health care and hence access to the most appropriate treatment regardless of cost [35].

## Conclusions

This study showed that the quality of life of parents of polyhandicapped individuals is severely deteriorated. The heaviest QoL determinants were the financial and health issues and the psycho-behavioural aspects. Because many of these determinants might be amenable, these findings should help health care workers and health decision-makers to implement specific and appropriate interventions.

## Supporting information

S1 Checklist Strobe checklist. (PDF)

S1 File Table A. Parents’ quality of life and characteristics of the parents (univariate analysis), Table B. Parents’ quality of life and PLH individuals’ characteristics, Table C. Parents’ quality of life and their social environment and healthcare satisfaction.
(PDF)

S1 Table Factors modulating quality of life in accordance with the mother/father status (using Generalized Estimating Equations models). (PDF)
