# Supplementary material for: Impact of severe polyhandicap on parents’ quality of life: A large French cross-sectional study
Source: PLoS One. 2019 Feb 4;14(2):e0211640. doi: 10.1371/journal.pone.0211640 (PMC6361449; doi:10.1371/journal.pone.0211640)
Supplement: S1 File — Table A. Parents’ quality of life and characteristics of the parents (univariate analysis), Table B. Parents’ quality of life and PLH individuals’ characteristics, Table C. Parents’ quality of life and their social environment and healthcare satisfaction. (PDF) [file pone.0211640.s002.pdf]

Table A. Parents' quality of life and characteristics of the parents (univariate analysis)

		Physical	Psychological	Social	Environ.
**1. Sociodemographics**					
Age	R	-0.07	0.016	0.03	0.13
	p-value	0.21	0.78	0.55	**0.014**
Nature of relationship	Mother	62.5±20.2	62.6±17.3	62.6±20.2	59±16.6
	Father	67.9±18.3	67.6±15.2	63.3±19.2	61.1±15
	p-value	**0.01**	**0.004**	0.75	0.21
Marital status	Not single	65±18.4	64.1±16	62.9±19.7	60±15.1
	Single	62.8±23.1	65.3±18.7	63.2±20.2	57.9±18.2
	p-value	0.39	0.54	0.89	0.18
Couple with the other parent	Yes	64±18.1	63.5±15.8	61.5±20	60±15.3
	No	65.8±21.8	66.1±17.9	65.2±19.7	59.6±16.8
	p-value	0.39	0.14	0.08	0.78
Number of children at home	R	-0.02	0.02	-0.009	-0.04
	p-value	0.97	0.72	0.87	0.48
Other handicapped person at home	Yes	57.8±22	61.1±17.5	60.8±21.2	54.6±15.7
	No	65.5±19.1	65.2±16.4	63.4±19.4	60.7±15.7
	p-value	**0.01**	0.112	0.39	**0.01**
Educational level	<12 years	62.2±21.5	63.7±17.2	62.5±18.8	57.3±16.6
	≥12 years	66.9±17.1	65.7±15.6	63.7±20.2	62.4±15
	p-value	**0.018**	0.25	0.56	**0.002**
Occupational status	Worker	69.8±16	66.9±16	64.5 ±20.2	62.4 ±.1
	Not worker	58.8±21.5	62±16.6	61.8±18.6	57±15.3
	p-value	**<0.0001**	**0.003**	0.192	**0.001**
Self-perceived financial status	Not difficult	67.7±17.6	67.3±14.9	64.8±18.9	63.6±13.8
	Difficult	55.7±22.2	57.1±18.5	58.7±20.8	49.2±17
	p-value	**<0.0001**	**<0.0001**	**0.008**	**<0.0001**

Presence of the patient at home per month	≥7 nights	63.9±18.8	63.5±16.7	59.2±20.5	57.5±14.9
	<7 nights	64.9±21	65.5±16.8	67.9±17.7	62.6±17
	p-value	0.62	0.24	**<0.0001**	**0.002**
**2. Health**					
Personal chronic disease(s)	Yes	53±20.6	60.6±17	57.6±21.8	55.5±16.8
	No	69.9±16.7	66.4±16.1	65.4±18.3	61.8±15.2
	p-value	**<0.0001**	**0.001**	**0.001**	**<0.0001**
Hospitalization during the last 2 years	Yes	54.2±21.2	59.9±16.7	58.1±20.6	55.6±16.8
	No	67±18.5	65.7±16.5	64±19.6	60.8±15.7
	p-value	**<10-3**	**<10-3**	**0.024**	**0.012**
**3. Psycho-behavorial characteristics**					
Anxiety-mood score (1-10)*	R	-0.45	-0.49	-0.40	-0.33
	p-value	**<0.0001**	**<0.0001**	**<0.0001**	**<0.0001**
Coping	Social support	-0.01	0.11	0.22	0.05
	p-value	0.71	**0.03**	**<0.0001**	0.29
	Problem solvings	0.20	0.34	0.28	0.26
	p-value	**<0.0001**	**<0.0001**	**<0.0001**	**<0.0001**
	Avoidance	-0.11	-0.24	-0.04	-0.04
	p-value	**0.02**	**<0.0001**	0.44	0.34
	Positive thinking	0.20	0.40	0.28	0.18
	p-value	**<0.0001**	**<0.0001**	**<0.0001**	**<0.0001**

* 1 absence to 10 very important disorder

Table B. Parents' quality of life and patients' characteristics

		**Physical**	**Psychological**	**Social**	**Environ.**
Age	R	-0.05	-0.001	0.09	0.11
	p-value	0.25	0.97	0.06	**0.02**
Gender	Women	65.8±19.2	67.5±16	66.7±19.1	62.2±15.7
	Man	63.2±20.2	61.9±16.9	59.8±19.8	57.7±16
	p-value	0.19	**0.001**	**0.001**	**0.007**
Severity*	Less severe	65±19.9	65.7±16.1	64.4±19.2	60.2±15.6
	Severe	63.7±19.7	63.4±17.1	61.8±20.3	59.4±16.3
	p-value	0.5	0.17	0.21	0.6
Stability**	Stable	63.9±20	64.7±16.6	63±19.9	60.3±16.4
	Unstable	65.2±19.4	63.8±16.9	63.1±19.7	58.5±15
	p-value	0.58	0.62	0.94	0.31
Medical devices***	Yes	64.6±19.4	63.6±16.6	63.4±19.1	59.5±16.2
	No	64±20.1	64.8±16.9	62.4±20	59.7±16
	p-value	0.79	0.48	0.65	0.91

* Severe case: association of motor handicap, IQ <25, FIM<=20, and GMFCS IV/V

**Unstable case: recurrent pulmonary infections and/or drug resistant epilepsy

*** At least one of the following list: invasive mechanical ventilation, non-invasive mechanical ventilation, tracheotomia, nasogastric tube, gastrostomy, permanent urinary probe, cerebrospinal fluid derivation, and central venous catheter

Table C. Parents' quality of life and their social environment and healthcare satisfaction (N=394)

	Physical	Psychological	Social	Environ.
**1. Parents' social environment**				
Family relationship preservation				
Yes (350)	64.5±19.1	65.1±15.8	64.1±18.9	60.3±15.2
No (35)	63.4±25.9	57.1±23	51±25.3	54.7±22.4
p-value	0.8	**0.052**	**0.005**	0.15
Patient presence during family celebrations				
Yes (286)	66.1±19	65.2±16.7	62.4±20.4	60±15.8
No (99)	60.7±20.5	63.1±16.1	65.4±17.2	59.8±16.4
p-value	**0.02**	0.28	0.20	0.89
Social network related to PLH				
Yes (135)	60.6±17.6	62.4±15	63.2±20	59.7±12.9
No (245)	65.8±20.4	64.7±17.5	62±20.2	59.6±17
p-value	**0.03**	0.25	0.63	0.95
Social network not related to PLH				
Yes (283)	66.8±18.2	66.1±15.6	65.7±17.8	62.1±14.8
No (91)	56.8±22.7	59.1±19.4	53.5±23	52.1±18
p-value	**<0.0001**	**0.002**	**<0.0001**	**<0.0001**
PLH associative community implication				
Yes (77)	62.7±17.3	65.7±13.7	62.8±18.4	60.6±13.9
No (311)	64.9±20.3	64.2±17.4	62.8±20.2	59.5±16.6
p-value	0.40	0.42	1	0.61
**2. Parents' healthcare system satisfaction**				
Medical information of the patient				
Rather satisfied (215)	65.6±20.2	65.8±16.4	65±18.9	61.9±15.6
Rather not satisfied (171)	63±19.1	62.4±17.1	59.9±20.9	56.9±16.3
p-value	0.21	**0.050**	**0.013**	**0.002**
Global management of the patient				
Rather satisfied (250)	65.8±19.5	65.8±16.6	65.7±18.9	61.4±15.6
Rather not satisfied (138)	61.8±20	62±16.7	58.3±20.4	56.9±16.5
p-value	**0.056**	**0.03**	**<0.0001**	**0.01**
Quality of care for the patient				
Rather satisfied (266)	65.3±19	65.7±16.6	64.8±18.6	60.9±15.7
Rather not satisfied (123)	62.3±21	61.4±16.7	58.6±21.8	57.3±16.5
p-value	0.15	**0.02**	**0.009**	**0.03**
Familial caregivers services				
Rather satisfied/ satisfied (212)	65.7±19.7	66.9±16.3	67.1±19.4	61.7±15.7
Very unsatisfied/not satisfied (170)	63±19.6	61.7±16.9	57.9±19.2	57.4±16.3
p-value	0.17	**0.003**	**<0.0001**	**0.01**
