# Supplementary material for: Impact of severe polyhandicap on parents’ quality of life: A large French cross-sectional study
Source: PLoS One. 2019 Feb 4;14(2):e0211640. doi: 10.1371/journal.pone.0211640 (PMC6361449; doi:10.1371/journal.pone.0211640)
Supplement: S1 Table — (PDF) [file pone.0211640.s003.pdf]

S1 table. Factors modulating quality of life in accordance with the mother/father status (using Generalized Estimating Equations models)

	**Physical**				**Psychological**				**Social**				**Environ.**			
	**Mothers**		**Fathers**		**Mothers**		**Fathers**		**Mothers**		**Fathers**		**Mothers**		**Fathers**	
	**β**	**p**	**β**	**p**	**β**	**p**	**β**	**p**	**β**	**p**	**β**	**p**	**β**	**p**	**β**	**p**
**1. Parents' variables**																
Age of the parent													-0,008	0,966	0,143	0,371
Marital status (0 single, 1 couple)													-2,224	0,301	-4,523	0,095
Couple initial of parents (0 yes, 1 no)					1,715	0,286	-2,76	0,280	6,471	**0,011**	-3,969	0,122				
Educational level (0 low, 1 high)	-3,456	0,1	1,446	0,606									-5,791	**0,007**	-0,617	0,805
Financial status (0 not difficult, 1 difficult)	3,748	0,156	10,284	**0,004**	7,382	**0,001**	6,444	**0,02**	4,713	0,111	-1,561	0,596	8,546	**0,001**	12,525	**$<10^{-3}$**
Occupational status (0 worker, 1 not worker)	9,137	**$<10^{-3}$**	3,454	0,267	2,513	0,154	-0,746	0,761	2,277	0,354	1,705	0,59	3,67	0,091	-0,977	0,751
Other handicapped person at home (0 no, 1 yes)	0,034	0,992	10,172	**0,003**	3,871	0,18	0,881	0,672					4,303	0,088	3,221	0,223
PLH individual at home (0 >= 7; < 7 nights)									-10,395	**0,001**	-6,259	0,074	-3,223	0,269	-8,059	**0,012**
Chronic disease (0 no, 1 yes)	11,712	**$<10^{-3}$**	10,075	**0,002**	-0,182	0,92	1,936	0,47	8,597	**0,001**	8,333	**0,014**	3,619	0,071	5,706	**0,048**
Hospitalization episode (0 no, 1 yes)	6,972	**0,004**	8,689	**0,038**	2,372	0,261	1,882	0,633	1,773	0,52	4,545	0,218	3,23	0,147	3,128	0,35
Anxiety-mood score	-2,129	**$<10^{-3}$**	-2,502	**$<10^{-3}$**	-1,911	**$<10^{-3}$**	-1,922	**$<10^{-3}$**	-1,818	**$<10^{-3}$**	-2,868	**$<10^{-3}$**	-1,133	**0,011**	-0,959	0,067
Coping Social support					0,099	**0,019**	0,06	0,338	0,241	**$<10^{-3}$**	0,148	**0,021**				
Problem solvings	0,131	**0,022**	0,142	0,063	0,111	**0,016**	0,19	**0,01**	0,047	0,402	0,228	**0,004**	0,193	**$<10^{-3}$**	0,139	0,083
Avoidance	-0,106	0,189	0,07	0,521	-0,293	**$<10^{-3}$**	-0,187	0,121								
Positive thinking	0,023	0,698	0,032	0,676	0,223	**$<10^{-3}$**	-0,005	0,934	0,186	**0,006**	-0,073	0,319	-0,011	0,852	-0,069	0,322
**2. PLH individuals' variables**																
Gender (0 boy/man, 1 girl/woman)					-1,021	0,581	-3,062	0,187	-4,018	0,094	-5,796	**0,031**	-1,502	0,463	-2,071	0,389
Age									-0,005	0,963	-0,002	0,99	0,138	0,5	-0,024	0,866

β beta standardized coefficient; Bold values: p-value <0.05
